# Modeling of loops in proteins: a multi-method approach

**DOI:** 10.1186/1472-6807-10-5

**Published:** 2010-02-11

**Authors:** Michal Jamroz, Andrzej Kolinski

**Affiliations:** 1 Laboratory of Theory of Biopolymers, Faculty of Chemistry, University of Warsaw, Pasteura 1, 02-093 Warsaw, Poland

## Abstract

**Background:**

Template-target sequence alignment and loop modeling are key components of protein comparative modeling. Short loops can be predicted with high accuracy using structural fragments from other, not necessairly homologous proteins, or by various minimization methods. For longer loops multiscale approaches employing coarse-grained *de novo *modeling techniques should be more effective.

**Results:**

For a representative set of protein structures of various structural classes test predictions of loop regions have been performed using MODELLER, ROSETTA, and a CABS coarse-grained *de novo *modeling tool. Loops of various length, from 4 to 25 residues, were modeled assuming an ideal target-template alignment of the remaining portions of the protein. It has been shown that classical modeling with MODELLER is usually better for short loops, while coarse-grained *de novo *modeling is more effective for longer loops. Even very long missing fragments in protein structures could be effectively modeled. Resolution of such models is usually on the level 2-6 Å, which could be sufficient for guiding protein engineering. Further improvement of modeling accuracy could be achieved by the combination of different methods. In particular, we used 10 top ranked models from sets of 500 models generated by MODELLER as multiple templates for CABS modeling. On average, the resulting molecular models were better than the models from individual methods.

**Conclusions:**

Accuracy of protein modeling, as demonstrated for the problem of loop modeling, could be improved by the combinations of different modeling techniques.

## Background

Comparative modeling remains the most dependable and routinely used method for protein structure prediction [1,2]. The alternative term of homology modeling is frequently used. That is because the identification of a structural template (or templates) is typically based (although not always) on the homology relation between the target protein and the templates, which is usually reflected by a certain level of sequence similarity. When a template is being identified by some advanced Fold Recognition (FR) techniques, it is sometimes possible to identify templates that are structurally similar to the target without any obvious homology relations. This could be a genuine case of convergent evolution or (more frequently) the case when remote homology just can not be detected. Template free, *de novo *structure prediction is much more difficult and less dependable, although a steady progress is observed in this area of computational biology [3,4]. Most contemporary methods for *de novo *structure predictions heavily depend on certain aspects of evolutionary relationships between protein sequences and structures. The evolutionary methods are essential for the derivation of statistical potentials for *de novo *modeling and/or are employed in various strategies for extracting structure building blocks from known protein structures [5,6].

Classical homology modeling consists of three steps. First, a template for modeling needs to be identified and sequence alignment between the template and target sequences has to be generated. Usually, template identification is performed by certain standard tools, such as PSI-BLAST, and the resulting alignment is subsequently rectified by other tools and eventually by manual expert corrections. Remote templates can also be identified by FR procedures [7]. With the decreasing level of sequence similarity, which implies increasing evolutionary distance and thereby increasing structural differences between the template and the target, alignments become more and more ambiguous. Accuracy of classical comparative modeling heavily relies on the fidelity of the template-target alignment.

In the second stage the aligned fragments of templates are used to generate the corresponding fragments of the target structure. In the simplest case of a single template only, this step reduces to mere copying the template coordinates according to the alignment. In the case of multiple templates a consensus scaffold could be built, for instance via the distribution of the spatial restrains read from the templates, as it is implemented in the MODELLER method [8]. The key component of this stage of modeling is construction of loop regions that are frequently missing in the template scaffold. In certain newer approaches to comparative modeling the entire structure of the target is built using templates as sources of restraints of various types [3,9]. The main aim and challenge of such approaches is to be able to build a model of the target structure which is more similar to the true structure of the target than to any of the templates used, especially for distant homology based modeling.

The third, and final, stage of modeling is structure refinement which involves repacking the side chains and energy minimization of the entire structure [10].

The above scheme, or its variants, of comparative modeling remains the best choice when significant fragments of the alignment are error-free, which is usually the case in the range of high level sequence similarity (e.g. 40%, or more, of identical residues in the alignment). In the "twilight zone" of low sequence similarity the alignments contain significant errors. These could be sometimes corrected by building a multi-template consensus modeling scaffold [11]. Alternatively, it is possible to design a completely different modeling schemes, in which the alignment is built simultaneously to the actual modeling process [12].

In this paper we address the issue of loop modeling, separating it from the alignment problem. The test set of proteins with missing loops consists of two sub-sets. The first subset, containing missing loops of 4-12 residues, has been taken from a recent work by Rossi, et al., excluding the cases of incomplete chains in the corresponding PDB entries [13]. The work provides a comprehensive comparison of loop modeling performance of the most popular comparative modeling software. The loop database employed in the work of Rossi was adapted from a compiled loop database assembled by Jacobson et al [13,14]. Additionally, the database used in this work was expanded with cases of much longer loops, up to 25 residues (the second sub-set). The database covers all the structural classes of proteins, with 186 internal loops of various length. The expanded range of the modeled loop lenghts addresses the possibility of the extension of the range of applicability and accuracy of challenging instances of comparative modeling. Four methods of loop modeling are compared in this work: MODELLER, ROSETTA, CABS and a combination of MODELLER with CABS. Since MODELLER is a commonly accepted reference standard in comparative modeling, the results are qualitatively (although indirectly) comparable with other approaches [13,15-20]. It should be noted that MODELLER is representative software for distance geometry and energy minimizations, while ROSETTA and CABS employ knowledge-based free search of a discretized conformational space. Thus, the comparison given in this paper should provide additional insights into the range of applicability of these qualitatively different approaches to protein molecular modeling. Previous computational experiments with the reconstruction of missing fragments of protein structures indicated that the coarse grained models (an early version of CABS and two other modeling tools based on similar principles) performed relatively well in the range of large fragments [21]. At this point we would like to present a comprehensive evaluation in a wide range of loop modeling instances.

## Results

For a representative test set of protein structures with missing loop fragments the loop reconstruction procedure was executed using MODELLER, ROSETTA, CABS and the MODELLER-CABS hybrid modeling pipeline. The test set is summarized in Table 1. Modeling procedures are described in the Methods section. The test proteins represent various structural classes, including mainly helical, beta and alpha/beta structures. All test structures are of high quality with resolution of at least 2 Å and the average temperature factor lower than 35. The missing loops are representative, and they are exposed to the solvent or partially buried, connecting various elements of the secondary structure. The modeled loops span a wide range of lengths, from 4 to 25 residues. This is a range that is relevant for standard comparative modeling. In several proteins more than one loop is modeled. In some cases the modeled loops can interact with one another, which can have some influence on the performance of respective methods.

**Table 1:** Protein codes and loop locations of test set of protein

Protein codes and loop locations of test set of protein.	
**loop length**	**PDB codes and loop ranges**

4	7rsa 47-50, 4gcr 116-119, 2tgi 72-75, 2exo 161-164, 1xif 82-85, 1tml 42-45, 1tib 46-49, 1thw 194-197, 1rcf 111-114, 1ppn 42-45, 1plc 74-77, 1pbe 117-120, 1nfp 37-40, 1frd 59-62, 1cbs 21-24, 1ads 99-102, 1aaj 82-85

5	7rsa 75-79, 2hbg 37-41, 2cmd 188-192, 1vcc 63-67, 1tml 147-151, 1tca 157-161, 1sbp 181-185, 1prn 187-191, 1noa 88-92, 1nfp 95-99, 1nar 56-60, 1kuh 37-41, 1hbq 158-162, 1hbg 19-23, 1frd 83-87, 153l 131-135

6	5p21 104-109, 3pte 256-261, 3pte 131-136, 2ayh 81-86, 1tca 94-99, 1tca 38-43, 1rge 73-78, 1noa 25-30, 1mrp 233-238, 1gca 100-105, 1ede 180-185, 1cbs 66-71, 1brt 253-258, 1brt 174-179, 1ads 150-155, 1ads 149-154

7	5p21 83-89, 2pth 95-101, 1tml 20-26, 1tca 132-138, 1php 135-141, 1mbd 17-23, 1lif 64-70, 1iab 142-148, 1hbg 46-52, 1gca 196-202, 1edg 309-315, 1dad 116-122, 1brt 226-232, 1bkf 64-70, 1ads 186-192

8	2ayh 194-201, 1tml 187-194, 1thw 18-25, 1prn 150-157, 1nwp 84-91, 1nls 97-104, 1nar 192-199, 1hbq 31-38, 1arb 136-143, 1alc 34-41, 1ads 274-281

9	3pte 107-115, 2ayh 169-177, 1xnb 133-141, 1xnb 116-124, 1php 91-99, 1nls 131-139, 1ede 257-265, 1arb 168-176, 1aac 58-66

10	7rsa 87-96, 7rsa 33-42, 7rsa 110-119, 2cmd 57-66, 1whi 47-56, 1tca 23-32, 1scs 65-74, 1ppn 190-199, 1plc 42-51, 1mrj 173-182, 1ixh 84-93, 1gvp 49-58, 1fkf 63-72, 1arb 41-50, 1amp 181-190, 1ads 171-180, 1ads 170-179, 135l 18-27

11	3pte 91-101, 2pth 8-18, 1rcf 122-132, 1ixh 120-130, 1dad 42-52, 153l 154-164

12	2ayh 21-32, 1ixh 160-171, 1bkf 9-20, 1arb 74-85, 153l 98-109

16	1tml 73-88, 1tml 219-234, 1tca 184-199, 1rge 37-52, 1prn 106-121, 1nar 10-25, 1iab 136-151, 1frd 33-48, 1edg 233-248, 1edg 167-182, 1brt 57-72, 1amp 98-113, 1ads 210-225

18	1tml 73-90, 1tml 219-236, 1tca 184-201, 1prn 106-123, 1nar 10-27, 1iab 136-153, 1byt 807-824, 1byt 700-717, 1byt 359-376, 1byt 230-247, 1bst 57-74, 1bst 129-146, 1b57 209-226, 1awj 2-19, 1amp 98-115, 1ahj 101-118, 1ads 210-227, 1acc 36-53, 1acc 183-200

20	1br4 390-409, 1br4 349-368, 1br4 291-310, 1br2 246-265, 1azx 362-381

22	1tml 219-240, 1prn 106-127, 1nar 10-31, 1kk7 291-312, 1jez 117-138, 1itk 179-200, 1itk 157-178, 1e04 351-372, 1clq 380-401, 1br4 71-92, 1br4 256-277, 1b3k 322-343, 1aoa 182-203

23	1nfb 253-275, 1lzj 2-24, 1izl 21-43, 1i50 46-68, 1dzg 367-389

24	1uoz 224-247, 1mnd 277-300, 1miu 93-116, 1i19 415-438, 1hfb 86-109

25	2hs0 319-343, 2gah 437-461, 2fqf 293-317, 2e4y 311-335, 1zba 16-40, 1tml 219-243, 1qme 127-151, 1prn 106-130, 1kmh 117-141, 1eah 247-271, 1dms 596-620, 1dhx 376-400, 1dhx 11-35

Using MODELLER, we generated 500 examples of individual loop regions, which were subsequently ranked by the DOPE statistical potential [22]. Top ranked means the highest rank, while the "best" result means a structure that is closest to the actual experimental, structure of the loop. Similarly, ROSETTA models were ranked with ROSETTA potentials. CABS modeling provides a trajectory containing several hundred instances. These were subject to the clustering procedure. Interestingly, in most cases the medoid from the entire simulation was closer to the true structure than the largest clusters' medoids. This suggest very good convergence of CABS simulations. Consequently, the medoid structures were reported as the top-ranked models.

**Figure 1:**
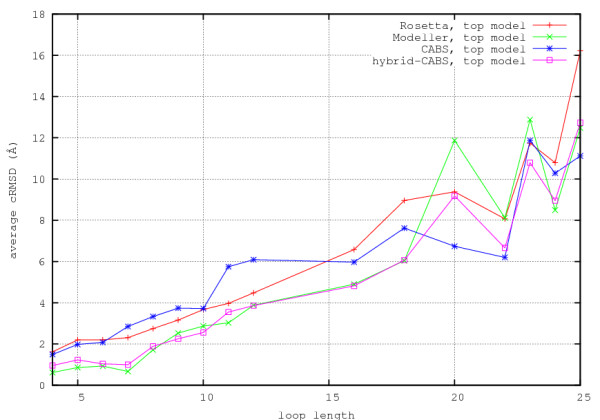
**Plots of average cRMSD of loops versus loop length**. Plots of average cRMSD of loops versus loop length. Best and top models generated by MODELLER, ROSETTA and CABS.

**Figure 2:**
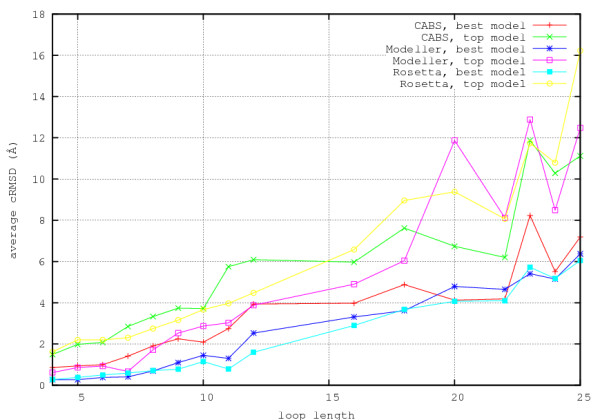
**Plots of average cRMSD of loops versus loop length**. Plots of average cRMSD of loops versus loop length. Top models generated by various modeling procedures, including MODELLER-CABS hybrid method (see the text).

The statistics of the results is shown in Figure 1, in which the loops of a given length are described by average values of cRMSD of the loop fragments (coordinate Root-Mean-Square Deviation) from the corresponding crystallographic structures. To extract the loop cRMSD values protein structures without the modeled loop fragments were superimposed and the deviation was computed only for the loops. In the entire text the values of cRMSD are reported for C*α *traces only. Corresponding data for all atom structures are essentially the same. The plots in Figure 1 clearly show two major trends. The first is obvious: with the increasing size of loops the average accuracy of modeling decreases. The second trend indicates, as expected, that the distribution of the quality of models, as measured by the difference between the best model and the top ranked models is much larger for MODELLER and ROSETTA as compared with CABS. For very long loops (20 and more residues) CABS results are on average better than for MODELLER and ROSETTA. The hybrid-CABS modeling takes advantages of different methods. Using top10 models generated by MODELLER the new method leads to results as good as MODELLER for short loops and noticeably better models in the range of long loops. When comparing with original CABS simulations the hybrid-CABS is much more accurate for short loops. This is illustrated in Figure 2. The results with hybrid-CABS show that there is always an added value in combining different modeling methods.

**Figure 3:**
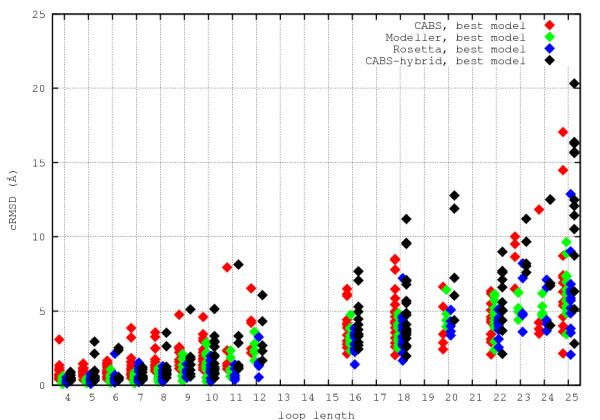
**Best loop models cRMSD**. Distribution of best loop models cRMSD for different modeling procedures.

**Figure 4:**
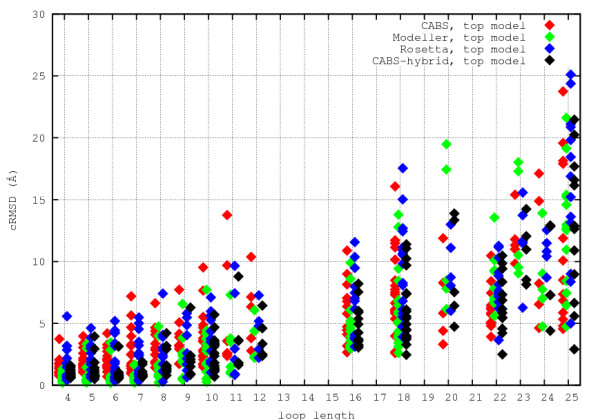
**Top ranked loop models cRMSD**. Distribution of top ranked loop models cRMSD for different modeling procedures.

Figure 3 and Figure 4 show the distributions of cRMSD for 186 cases studied. The distribution is quite broad, especially for longer loops. Use of distinct modeling techniques increases chances for obtaining good quality models. Unfortunately, all methods produce results of scattered quality. The problem how to identify the cases for which the models are of good accuracy remains unsolved.

## Discussion

**Figure 5:**
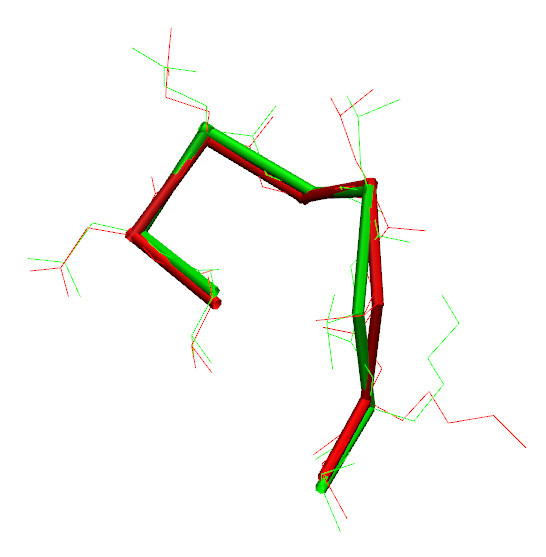
**Superimposition**. Sidechains of 149-154 loop from 1ads crystallographic structure (red) with superimposed CABS (cRMSD 0.70 Å) model (green).

**Figure 6:**
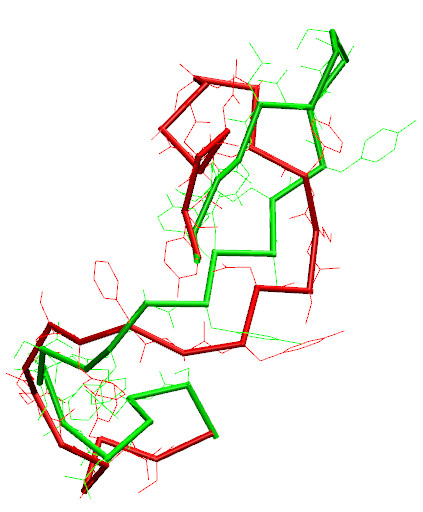
**Superimposition**. Loop fragment 106-130 of 1prn crystallographic structure (red) superimposed with CABS (cRMSD 4.80 Å) model (green).

The loop modeling exercise described in this paper separates the two fundamental problems of comparative modeling: target-template alignment and modeling of missing fragments. Ideal alignments have been assumed and the excised loops reconstructed and compared with the native structures (cRMSD of the reconstructed loops after the superposition of the fixed parts of templates and models). As expected, MODELLER and ROSETTA proved to be more accurate for short loops, while CABS models were better for longer loops (see the compilation of cRMSD values for different ranges of loop sizes, shown in Table 2(two-sample paired t-test, data in Additional file 1)), although the difference is small. In spite of the coarse-grained character of the method, the models from CABS allow for the meaningful reconstruction of the side chain details for shorter, and therefore more accurately predicted, loops (see Figure 5). The predicted side-chains conformations, shown in Figure 5, are of crystallographic accuracy, except for the tail portion of one side-chain. For longer loops the side chains are less accurate and their native-like conformations and interaction patterns are observed only for the best models. Figure 6 shows a typical situation for the loops from the range of accuracy of 4-6 Å. In such cases the side chains are approximately at proper positions, although their conformations on the atomic level are not reproduced. Finally, it should be noted that the simulation results from CABS could be used for the analysis of loop dynamics. In recent publications we have shown that isothermal trajectories from CABS, executed at the folding transition temperature, reproduce folding mechanisms of small proteins very well [23,24]. Thus loop mobility could also be modeled. In order to obtain the best possible model of the lowest energy structures, in the present study we used Replica Exchange Monte Carlo. Thus, the dynamics of the system is artificial. Obviously, isothermal simulations could be performed for the models obtained, leading to meaningful description of loop mobility. This was, however, beyond the scope of the present work.

**Table 2:** Summary of the average results from both modeling techniques.

Average cRMSD (in Å)				
**Loop range**	**CABS top (best)**	**Modeller top (best)**	**Rosetta top (best)**	**Modeller-CABS hybrid top (best)**

4-6	**1.84 (0.93)**	**0.80 (0.31)**	**2.00 (0.38)**	1.07 (0.66)

7-12	**3.83 (2.13)**	2.20 **(1.10)**	**3.21 (0.89)**	2.23 (1.75)

16-25	8.11 **(5.23)**	8.39 **(4.54)**	**10.02 (4.31)**	7.87 (7.07)

Average coordinate Root Mean-Square Deviation (cRMSD) from crystallographic structures in Ångstroms. Bold fonts indicates statistically relevant differences between individual methods and Modeller-CABS hybrid method (two-sample paired t-test, data in Additional file 1).

## Conclusions

In this work we have shown that *de novo *protein loops modeling using ROSETTA and CABS-based software is complementary to the classical modeling with MODELLER, the golden standard of comparative modeling. The proposed hybrid modeling pipeline, where ten top ranked (according to DOPE statistical potential) MODELLER models are used as templates for CABS, allows for meaningful loop modeling for a broad range of loop length. The hybrid MODELLER-CABS method takes advantage of the local accuracy of MODELLER structures and the efficient sampling of local free-energy minima by CABS. The hybrid-CABS method described in this work extends the applicability range of protein comparative modeling. Further increase of accuracy for large loops will require better ranking of resulting models. Model-ranking in the range of moderate- and low-resolution computational structures remains a challenging problem for the entire structure-prediction field. In this case, a small step in this direction was performed by a combination of different modeling techniques.

## Methods

The dataset employed in this work is summarized in Table 1. The cases of shorter loops, up to 12 residues, are taken from the work of Rossi et al. who used a loop database developed by Jacobson et al. [13,14]. The longer loops were selected from the same protein structures as continuous fragments of coil structures, according to the DSSP definition of secondary structure. Dangling ends are excluded from our test, similarly as it was done by others. Dangling ends are frequently structurally poorly defined, and therefore the results of their simulations are difficult to interpret. The dataset is available for download (Additional file 2).

### Loop modeling with MODELLER and ROSETTA

All loops were first modeled using MODELLER, version 9v5, and the model-loop procedure [1]. The 500 resulting models were ranked using DOPE statistical potentials. Subsequently, loop modeling was repeated using ROSETTA software, leading to 500 independent models, ranked by the ROSETTA force field [25]. The description of the CABS modeling tool and the procedure employed in present study is provided below.

### CABS modeling software

CABS is a versatile modeling tool, based on the coarse graining of polypeptide conformational space and knowledge-based force field. Applications of CABS include protein structure prediction (from comparative to template-free modeling), prediction of protein folding mechanisms and flexible modeling of macromolecular assemblies [3,23,24,26]. Technical details of CABS design and software are provided elsewhere [27]. At this point, for the reader's convenience, we provide only an outline of the most essential features. The CABS (C-alpha, C-beta, and Side chain) representation of protein conformational space employs a united residue approach. A single amino acid is represented by four pseudo-atoms: centered on the alpha carbon, on the beta carbon, in the center of mass of the side chain (where applicable) and an additional pseudo-atom located in the center of the virtual C*α*-C*α *bond. The C*α *pseudo atoms are restricted to vertices of regular cubic lattice with the lattice spacing equal to 0.61 Å. Due to allowed fluctuations of the C*α*-C*α *distance around the canonical value of 3.78 Å the set of possible representations of this virtual bond consists of 800 lattice vectors. Thus, serious lattice artifacts could be safely ignored. The accuracy of the C*α*-trace projection onto this lattice is in the order of 0.35 Å. On the other hand, lattice representation smoothens the model energy landscape and speeds -up computation by using pre-computed local conformational transitions which require simple references to hashing tables instead of computing trigonometric transformations, as would be necessary in an otherwise equivalent continuous space model. Coordinates of other pseudo-atoms are off-lattice and are defined in the reference frame provided by the C*α *trace. Again, these coordinates are pre-computed and stored in simple reference tables, in which the two indices (range of 1-800, each) encode the conformation of three consecutive alpha carbons. It is assumed that coordinates of such fragments define positions of the side chain for the central residue.

Conformational updates include various local transformations, controlled by a pseudo random mechanism. There are single C*α *moves, two, three and four C*α *fragment transitions and small displacements of larger (4-22 residue) fragments. Update of a single C*α *position involves side chain updates of the central and two neighboring residues. The sampling scheme could be executed within a classical Metropolis Monte Carlo scheme (when isothermal dynamics is required) or using a Replica Exchange (REMC) protocol when equilibrium data are required only, as in the case of the present work.

The force field of CABS consist of several types of potentials, including the hard-core excluded volume of the main chain and C*β *atoms, generic (sequence independent) short-range protein-like biases, making the model chain behaving like a generic polypeptide chain, sequence-dependent short-range statistical potential, context-specific pairwise interactions of the side chain united atoms, with repulsive and attractive square-well potential, and finally, a model of main-chain cooperative hydrogen bond networks. The details of the force field could be found in earlier publications and the numerical data for the histogram-type potentials are available from the authors' homepage http://biocomp.chem.uw.edu.pl.

CABS allows for very straightforward implementation of restraints of various types. These may include soft biases towards predicted secondary structures and theoretically predicted side chain contacts, distance restraints read from templates for comparative modeling, restraints derived from sparse NMR data, etc.

### Loop modeling procedure with CABS

First, the template proteins (with excised loop fragments) are projected onto the CABS lattice, and the loop fragments are added in a random fashion. Then, the non-loop fragments of the original structures are used to read several hundreds of distance restraints, similarly to the procedures used in comparative modeling with CABS [3]. Subsequently, the starting structure is copied to 20 identical replicas for REMC simulations. During the REMC simulations temperatures of all replicas were gradually lowered, with a constant temperature distance between the replicas. Only the snapshots from the lowest temperature replica were stored in a pseudo-trajectory. Each simulation was repeated three times (with different streams of pseudo-random numbers), and the collated results were subject to final analysis. Trajectories were clustered using the K-means method. Also the medoids and the best observed structures from each trajectory were stored. It was observed that the centroids of the largest clusters were very close to the centroids from the entire trajectories. Thus, the trajectory medoid structures were reported as the top ranking models. For the top CABS structures the full atom molecular models were built using BBQ and SCWRL software [28,29]. Such a multiscale modeling strategy (from coarse-grained to all atom structures) proved very efficient in earlier applications of CABS software. Modeling of a single protein from the test set employed in this work using CABS protocol requires 10-15 hours of single LINUX box, which is similar to the cost of generating 500 structured by the ROSETTA method. Generation of 500 examples using MODELLER is 2-3 times faster.

### Hierarchical modeling with MODELLER and CABS

Analysis of preliminary modeling results led to an interesting observation: The distribution of the accuracy of the models generated by MODELLER and ROSETTA was significantly broader than the distribution of the quality of models generated by CABS.

The reason is that the models generated by MODELLER and ROSETTA are independent of one another, while the models from CABS are highly correlated along the simulation pseudo-trajectory. Consequently, the best models (among the 500 generated by MODELLER or ROSETTA) are usually considerably better than the top-ranked models. Unfortunately, the selection of the best models from a large set of decoys remains an unsolved problem for each of these methods. Taking the above into consideration, we designed a hybrid modeling pipeline that should take advantages of these methods. Namely, top ranked models from MODELLER (top 10) were used as structural templates for the derivation of distance restrains (including loop fragments) for modeling with CABS. It was expected that better local geometry of MODELLER structures and their diversity should improve sampling with CABS. The result of such an approach are reported as the CABS-hybrid simulations. Medoids (structures closest to the structural centroid from a pseudo-trajectory) were reported as the top ranked models. A similar modeling strategy was designed for a combination of ROSETTA and CABS. The accuracy of such an approach is similar to the accuracy of the aformentioned MODELLER-CABS hybrid. Since MODELLER is computationally less expensive than ROSETTA we present a benchmark only for the later combination.

## Authors' contributions

AK conceived the use of a combination of modeling techniques. MJ performed simulations and analysis of the results. AK drafted the manuscript and both authors read and approved the final version of the manuscript.

## Supplementary Material

Additional file 1 **Student t-test**. Results from two sample paired t-test of Table 2. Click here for file

Additional file 2 **Loop benchmark test set**. Database of 186 experimentally derived protein loop models used in the simulations. Click here for file
